# Allergen-induced CD11c + dendritic cell pyroptosis aggravates allergic rhinitis

**DOI:** 10.1186/s12964-023-01309-8

**Published:** 2023-10-10

**Authors:** Yue-Long Qiao, Ming-Wan Zhu, Shan Xu, Wo-Er Jiao, Hai-Feng Ni, Ze-Zhang Tao, Shi-Ming Chen

**Affiliations:** 1 Department of Otolaryngology-Head and Neck Surgery, Renmin Hospital of Wuhan University, 238 Jie-Fang Road, Wuhan, 430060 Hubei P.R. China; 2 Department of Otolaryngology-Head and Neck surgery, Affiliated Hangzhou First People’s Hospital, Zhejiang University School of Medicine, Hangzhou, Zhejiang 310006 P.R. China; 3 Institute of Otolaryngology-Head and Neck Surgery, Renmin Hospital of Wuhan University, 238 Jie-Fang Road, Wuhan, Hubei 430060 P.R. China

**Keywords:** Allergic rhinitis, Dendritic cell, GSDMD-N, Pyroptosis

## Abstract

**Background:**

Pyroptosis is crucial for controlling various immune cells. However, the role of allergen-induced CD11c + dendritic cell (DC) pyroptosis in allergic rhinitis (AR) remains unclear.

**Methods:**

Mice were grouped into the control group, AR group and necrosulfonamide-treated AR group (AR + NSA group). The allergic symptom scores, OVA-sIgE titres, serum IL-1β/IL-18 levels, histopathological characteristics and T-helper cell-related cytokines were evaluated. CD11c/GSDMD-N-positive cells were examined by immunofluorescence analysis. Murine CD11c + bone marrow-derived DCs (BMDCs) were induced in vitro, stimulated with OVA/HDM, treated with necrosulfonamide (NSA), and further cocultured with lymphocytes to assess BMDC function. An adoptive transfer murine model was used to study the role of BMDC pyroptosis in allergic rhinitis.

**Results:**

Inhibiting GSDMD-N-mediated pyroptosis markedly protected against Th1/Th2/Th17 imbalance and alleviated inflammatory responses in the AR model. GSDMD-N was mainly coexpressed with CD11c (a DC marker) in AR mice. In vitro, OVA/HDM stimulation increased pyroptotic morphological abnormalities and increased the expression of pyroptosis-related proteins in a dose-dependent manner; moreover, inhibiting pyroptosis significantly decreased pyroptotic morphology and NLRP3, C-Caspase1 and GSDMD-N expression. In addition, OVA-induced BMDC pyroptosis affected CD4 + T-cell differentiation and related cytokine levels, leading to Th1/Th2/Th17 cell imbalance. However, the Th1/Th2/Th17 cell immune imbalance was significantly reversed by NSA. Adoptive transfer of OVA-loaded BMDCs promoted allergic inflammation, while the administration of NSA to OVA-loaded BMDCs significantly reduced AR inflammation.

**Conclusion:**

Allergen-induced dendritic cell pyroptosis promotes the development of allergic rhinitis through GSDMD-N-mediated pyroptosis, which provides a clue to allergic disease interventions.

Video Abstract

**Supplementary Information:**

The online version contains supplementary material available at 10.1186/s12964-023-01309-8.

## Introduction

Dendritic cells (DCs) are the most powerful antigen-presenting cells (APCs) and are involved in a variety of immune disorders. The physiological functions of DCs, including presenting external antigens and inducing the differentiation of downstream T cells, play an important role in allergic diseases [1]. Allergic rhinitis (AR) is a chronic non-infectious inflammation of the nasal mucosa mediated by IgE that occurs in atopic individuals exposed to allergens. The incidence of AR is 10 ~ 40% worldwide [2], and while its pathogenesis is complex, the core process involves DC-mediated antigen presentation. How DCs, which are the source and mediator of allergic inflammation, affect the pathogenesis of AR in subjects in contact with allergens remains unclear.

Pyroptosis is an inflammatory caspase-mediated form of cell death [3, 4]. Briefly, the activation of inflammasomes in response to various infectious and immunological challenges commonly induces the activation of caspase-1 (canonical pyroptosis) or caspase 4/5/11 (noncanonical pyroptosis) [5, 6], leading to cell swelling and the leakage of intracellular proinflammatory substrates [7]. Notably, recent studies identified the pyroptosis executioner gasdermin D (GSDMD) as a substrate of both caspase-1 and caspase-11/4/5 [6, 8]. The cleavage of GSDMD by inflammatory caspases critically stimulates pyroptosis by releasing the cleaved GSDMD-N domain (GSDMD-N), which forms pores in the cell membrane, resulting in the secretion of the proinflammatory cytokines IL-1β and IL-18 and other severe inflammatory responses [9].

Pyroptosis has been shown to be involved in the development and progression of allergic diseases. In asthma, bronchial epithelial pyroptosis was shown to exacerbate airway inflammation and hyperresponsiveness via NLRP3 inflammasome activation and GSDMD cleavage [10]. Similarly, in AR, nasal epithelial pyroptosis was shown to play a role in promoting the development and progression of AR by enhancing the inflammatory response [11]. However, the pyroptosis of DCs, which play a key role in initiating adaptive immunity and are believed to be the centre of the immune system [12, 13], has not yet been reported.

Based on the report by Rathkey [14, 15], necrosulfonamide (NSA) was used as a GSDMD-N inhibitor to monitor pyroptosis-related protein levels and inflammatory changes in AR mice. Moreover, bone marrow-derived DCs (BMDCs) were cultured in vitro and stimulated with OVA to study the effect and mechanism of allergens on CD11c + BMDC pyroptosis. These cells were further cocultured with lymphocytes to study the effect of pyroptosis on BMDC function. We focused on whether GSDMD-N-mediated CD11c + DC pyroptosis plays a role in exacerbating AR and its underlying mechanisms.

## Materials and methods

### Animals

Female C57BL/6 mice (4–6 weeks old) were purchased from Beijing Weitonglihua Experimental Animal Technology and maintained at the Animal Experiment Centre of Renmin Hospital of Wuhan University (licence no.: SYXK (E) 2015-0027) under specific pathogen-free (SPF) conditions. The mice were housed at room temperature (approximately 18–22 °C) with a 12 h dark/light cycle and moderate humidity (approximately 50–60%). All mice had free access to food and water. The experiments described herein were approved by the Institutional Animal Care and Use Committee of Renmin Hospital of Wuhan University (licence no.: WDRM-20190509).

### Animal model

#### Murine model treated with a pyroptosis inhibitor (necrosulfonamide)

Six-week-old C57BL/6J mice were randomly divided into 3 groups (n = 8/group): normal group, AR group, and necrosulfonamide-treated AR group (AR + NSA group). The normal group was left untreated, and the other two groups were intraperitoneally injected with 100 µg of OVA (Sigma, USA) and 2 mg of Al(OH)_3_ (Pierce Chemical, USA) in a saline suspension for basic sensitization on Days 1, 3, 5, 7, 9, 11, and 13. On Days 15 to 28, mice in the AR group were intranasally challenged with OVA (10% in saline) once per day for 14 consecutive days, and mice in the AR + NSA group were intraperitoneally injected with NSA (20 mg/kg; Absin, China, #abs814352) 30 min before each OVA administration [14, 16].

#### Adoptive transfer murine model

Whole bone marrow cells were isolated from twenty-seven naive C57BL/6J mice and cultured in RPMI 1640 medium supplemented with GM-CSF (20 ng/mL) and IL-4 (20 ng/mL) at 37 °C in the presence of 5% CO_2_. On Days 3 and 5, the medium was refreshed. On Day 5, TNF-α (15 ng/mL) was added to further induce BMDC differentiation. On Day 6, BMDCs were harvested and incubated in 6-well plates in the presence of OVA protein (500 µg/mL) and/or NSA for 24 h. On Day 7, the cells were adoptively transferred to recipient mice for further analysis. Nine naive C57BL/6 mice were randomly divided into 3 groups (n = 3/group): the control group (BMDCs), OVA group (500 µg OVA-loaded BMDCs), and NSA group (5 mM NSA + 500 µg OVA-loaded BMDCs). On Days 0, 2 and 4, 1 × 10^7^ BMDCs (control group), 1 × 10^7^ OVA-loaded BMDCs (OVA group) or 1 × 10^7^ OVA (500 µg)-loaded BMDCs pretreated with 5 mM NSA (NSA group) in a total volume of 200 µL were injected i.v. (via the tail vein) into naive C57/BL6 mice. The mice were analysed 24 h after the last transfer.

### Immunofluorescence staining

Twenty-four hours after the last nasal administration in each group, 3 mice were randomly anaesthetized and killed, and the spleen, nasal bone and draining lymph nodes of the nose were collected. The nasal draining lymph nodes were collected according to the method described by Miyaga N et al. [17]. The tissues were fixed in paraformaldehyde. After 24 h, the draining lymph nodes of the nose were embedded with paraffin wax and prepared into sections. After 24 h, the nasal bone was placed into 10% EDTA decalcification solution for 2 weeks, and the decalcification solution was changed every 7 days. After 2 weeks, the nasal samples and spleen were embedded in paraffin wax and prepared into sections. Murine nasal and draining lymph node tissue samples were incubated with goat GSDMD-N (Affinity, China, DF13758) and CD11c (Servicebio, China, GB11059) antibodies. Murine splenic tissue samples were incubated with goat GSDMD-N (Affinity, China, AF4013), CD11c (Servicebio, China, GB11059), CD117 (Bioss, China, bs-2o717R), and ECP (Proteintech, China, 55338-1-AP) antibodies. Subsequently, the samples were incubated with donkey anti-goat IgG and Hoechst 33342 and then observed and imaged by confocal microscopy. The data were derived from three independent experiments.

### IL-1β, IL-18, OVA-specific serum immunoglobulin E (IgE) assays

Blood samples were harvested from the orbital venous plexus of anaesthetized mice at the time of death. Sera were isolated from blood samples by centrifugation for 15 min at 1000 ×g and stored at -80 °C until use. The animal sera were analysed using ELISA kits (IL-1β, #MU30369; IL-18, #MU30380; OVA-sIgE, #MU30065; Bioswamp, China). The data from three independent experiments are presented, and there were 8 mice in each group.

### Allergic symptom scores

The mice were monitored for allergic symptoms 30 min after the last OVA challenge. The frequencies of rubbing and sneezing were recorded, and scores were calculated as previously described [18, 19].

### Western blotting

Protein samples were prepared, and equivalent amounts of denatured protein were loaded onto 10% SDS‒PAGE gels. The proteins were electrotransferred onto PVDF membranes (GE Life, USA, #10600023), which were then blocked with 5% nonfat milk in TBST (20 mM Tris-HCl, 0.15 M NaCl, and 0.05% Tween-20, pH 7.5) for 1 h at room temperature. Then, the membranes were incubated with primary antibodies against GSDMD-N (Affinity, China, DF13758), NLRP3 (1:1000; Novus; NBP212446), Caspase-1 (1:1000; Servicebio, China, GB13483) and GAPDH (1:2000; Servicebio, GB11002) overnight at 4 °C. The membranes were then incubated with HRP-conjugated secondary antibodies (1:20,000) for 2 h at room temperature. Immunoreactive bands were visualized using an Immobilon western chemiluminescent HRP substrate (Millipore, USA) and quantified using ImageJ software. The data were derived from three independent experiments.

### Morphological observations of the nasal mucosa

Twenty-four hours after the last OVA challenge, the mice were euthanized. The noses were removed from mice after the facial skin was stripped. The fixed nasal bones were embedded in paraffin and sectioned, and haematoxylin and eosin (H&E) staining was used to identify nasal eosinophil infiltration. Periodic acid-Schiff (PAS) staining was used to identify the number of PAS-positive goblet cells. To analyse these two cell types, the average cell counts in 5 randomly selected fields were determined under a high magnification (400×). The counting method was described in our previous work [18, 19].

### Cell culture in vitro

#### Generation of murine BMDCs in vitro

Six-week-old SPF C57BL/6 mice were sacrificed by cervical dislocation under anaesthesia and soaked in a 75% ethanol solution for 10 min. The femurs and tibias were removed and washed with sterile PBS buffer to obtain a cell suspension. Red blood cells were lysed with RBC lysis buffer for 1 min, and bone marrow cells (BM cells) were collected by centrifugation. The BM cells were seeded in the centre of 6-well plates at 1 × 10^6^/ml in 3 ml of cell culture medium containing 20 ng/ml GM-CSF and 20 ng/ml IL-4 at 37 °C and incubated in a 5% CO_2_ incubator [20]. On Days 3 and 5, half of the liquid was discarded, and the cytokines were added in equal proportions [21]. On Day 5, TNF-α (15 ng/ml, R&D, 410-MT-010) was added to the culture media.

#### Treatment of BMDCs in vitro

The BMDCs were collected and divided into the control group (BMDCs, no treatment), low-concentration group (OVA 100 µg/ml or HDM 1 µg/ml), high-concentration group (OVA 500 µg/mL or HDM 10 µg/ml), and pyroptosis inhibitor group (500 µg/mL OVA + 5 µΜ NSA or 10 µg/ml HDM + 5 µΜ NSA). The latter three groups of cells (excluding the control group) were treated with the indicated factors on the 6th day, and related tests were carried out on the 7th day. In addition, programmed cell death detection kits (Annexin V + and 7-AAD +, Hangzhou Lianke Biotechnology Co., Ltd.) were used to examine the level of programmed cell death.

Wild-type C57BL/6 mice aged 8 weeks were sacrificed by cervical dislocation under anaesthesia. The spleen was dissected, washed and thoroughly ground on an ultraclean workbench, followed by the addition of red cell lysis buffer to dissolve red blood cells. After being washed with PBS, the cells were further filtered through a 300-mesh filter to finally obtain a single-cell suspension of splenic lymphocytes. BMDCs treated with different factors were cocultured with lymphocytes (1:10), and the relevant experiments were conducted after continuous culture for 48 h [20, 21].

### Flow cytometry

To analyse the maturity of BMDCs, the cells were cultured in vitro, harvested and adjusted to 1 × 10^6^ cells/ml. Then, single-cell suspensions (500 µl) were collected, blocked with sheep serum at 4 °C for 20 min and incubated with antibody cocktails including APC-CD11c (BD Pharmingen, USA, 561119), PE-CD80 (BD Pharmingen, USA, 561955), FITC-CD86 (BD Pharmingen, USA, 561962) and PerCP-Cy5.5-MHC II (BD Pharmingen, USA, 562363) at 4 °C in the dark for 30 min. After being washed twice, the single-cell suspensions were filtered three times with a 200 mesh filter and placed in new Eppendorf (EP) tubes for flow cytometric analysis.

To assess the effects of BMDC pyroptosis on T-cell numbers, BMDCs treated with the different factors were cocultured with lymphocytes for 48 h, and the cells and supernatants were collected by centrifugation. Single-cell suspensions were obtained, and CD4-FITC (BD, USA, 553046), PB450-T-bet (BD, USA, 563318), PB450-GATA3 (BD, USA, 563349), and Qdot655-RORγt (BD, USA, 564723) antibodies were added. The cells were incubated in the dark for 30 min at 4 °C. The single-cell suspension was washed twice with PBS and then filtered with a 200 mesh filter into a new EP tube for flow cytometric analysis. The changes in the supernatant levels of related cytokines, including IL-2, IFN-γ, IL-6 and IL-17 A, which are related to Th1/Th2/Th17 cells, were measured with a cytokine microsphere array kit. A Beckman flow cytometer (Beckman Coulter Inc.), FlowJo (Tree Star Inc.) and an FCAP Array (Soft Flow Inc.) were used to analyse the data, which were derived from three independent experiments.

### Real-time quantitative PCR

Total RNA was extracted from cell lysates using TRIzol reagent (Invitrogen), and cDNA was reverse transcribed using a RevertAid First Strand cDNA Synthesis Kit (Thermo Fisher Scientific, USA). Real-time quantitative PCR was performed using FastStart Universal SYBR Green Master Mix (Rox; Roche). First, the extracted RNA was reverse transcribed on a PCR instrument. The specific reverse transcription conditions were as follows: 25 °C for 300 s, 42 °C for 300 s, and 25 °C for 300 s. After being centrifuged, the cDNA was added to the PCR plate for PCR amplification according to the following reaction conditions: predenaturation at 95 °C for 30 s, denaturation at 95 °C for 15 s, annealing at 60 °C for 30 s, and extension at 72 °C for 45 s for 40 cycles in total. Then, the default setting of the instrument was followed (60℃→95℃, 0.5℃ every 5 s) [22]. The primers are shown in Table 1.

**Table 1:** The primers of Real-time quantitative PCR

**Name**	**Sequence (5′ –3′ )**
**T-bet**	
Forward	CCACCTGTTGTGGTCCAAGTTC
Reverse	CCACAAACATCTGTAATGCTTG
**GATA3**	
Forward	CCTCTGGAGGAGGAACGCTAAT
Reverse	GTTTCGGGTCTGGATGCCTTCT
**RORγt**	
Forward	GTGGAGTTTGCCAAGCGGCTTT
Reverse	CCTGCACATTCTGACTAGGACG

### Electron micrograph

When the BMDCs were cultured, cell climbing slices were added to the dishes, and the culture medium was discarded after the BMDC slides were prepared. The cell climbing slices were washed with PBS, fixed with electron microscopy fixative at room temperature for 2 h, and then transferred to a 4 °C refrigerator for storage. The fixed samples were rinsed 3 times with 0.1 M phosphate buffer PB (pH 7.4) for 15 min each. The samples were then fixed in the dark with 0.1 M phosphate buffer PB (pH 7.4) prepared with 1% osmic acid at room temperature for 1–2 h. Subsequently, the samples were washed three times with 0.1 M phosphate buffer PB (pH 7.4) for 15 min each time, 30-50%-70-80%-90-95%-100%-100% alcohol was successively added and incubated for 15 min each time, and isoamyl acetate was added and incubated for 15 min. Finally, the samples were dried in a critical point dryer, attached to the double-sided adhesive on the conductive carbon film and placed on the ion sputtering instrument sample table for approximately 30 s of gold spraying. The images were observed under a scanning electron microscope (Hitachi SU8100) [23].

### Statistical analysis

Statistical analysis was performed using SPSS software V.20.0 (IBM Corp., Armonk, US) and GraphPad Prism 9.0 (GraphPad Software, USA). The Shapiro–Wilk method was used to determine whether the data were normally distributed. The Levene method was used to determine the homogeneity of variance. Continuous variables with a normal distribution are described as the mean ± standard deviation. Student’s t test was used for comparisons between two groups. Comparisons between multiple groups were made using one–way analysis of variance, and Tukey’s test was used for pairwise comparisons. *P* values < 0.05 indicate significant differences.

## Results

### GSDMD-N-mediated pyroptosis was abnormally activated in CD11c + DCs

**Fig. 1:**
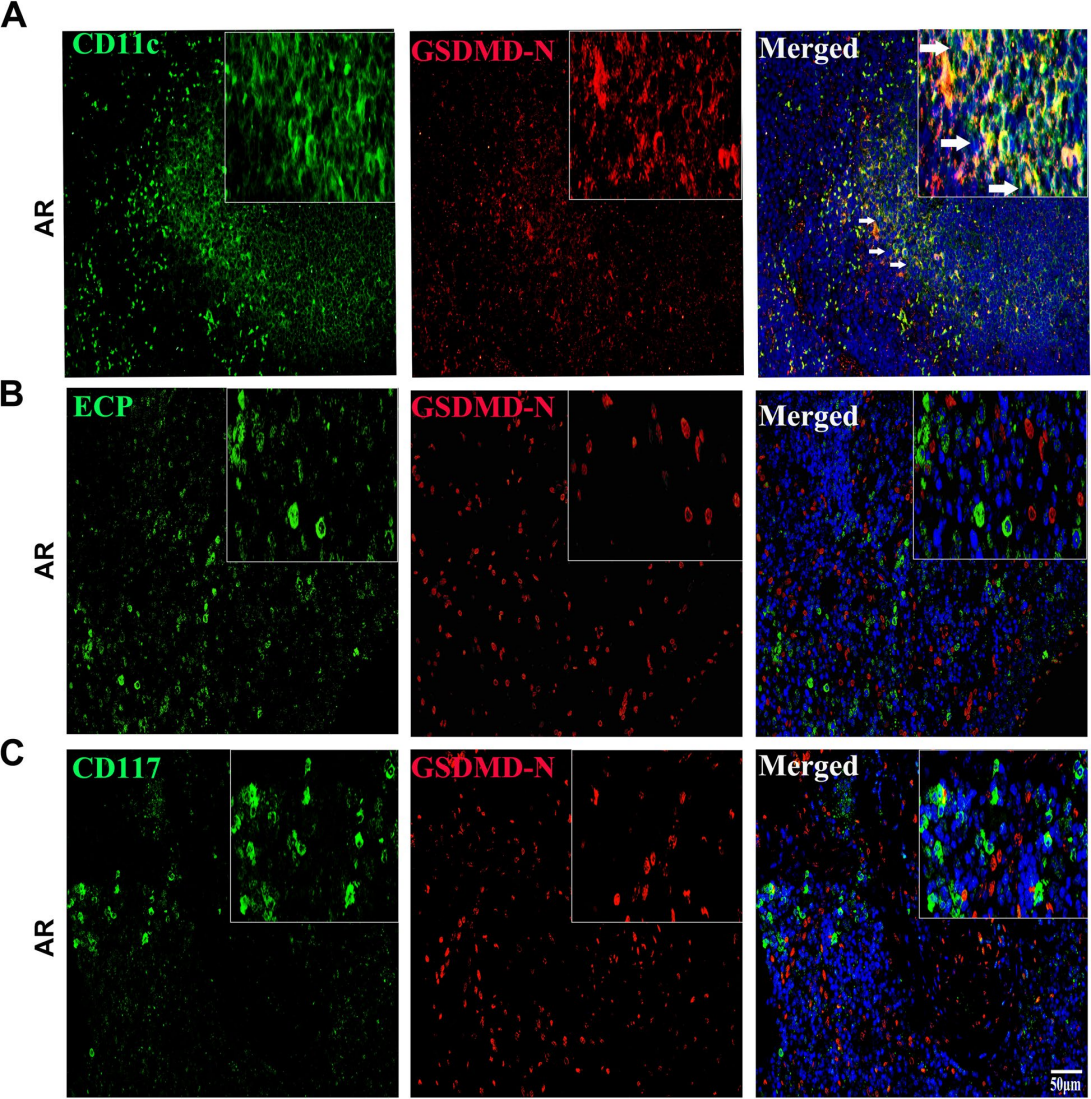
GSDMD-N-mediated pyroptosis was abnormally activated in CD11c + DCs. **A** ~ **C** Dual immunofluorescence analysis was performed with anti-GSDMD-N and anti-CD11c antibodies to detect CD11c + DCs, anti-GSDMD-N and anti-ECP antibodies were used to detect eosinophils, and anti-GSDMD-N and anti-CD117 antibodies were used to detect mast cells in mouse spleens

To study the main cell subsets involved in pyroptosis in AR mice, mouse spleen cells were examined. Immunofluorescence assays showed a high degree of GSDMD-N and CD11c colocalization in the spleen, and no obvious colocalization of GSDMD-N with ECP (eosinophil marker) or CD117 (mast cell marker) was observed (Fig. 1A ~ C). These results demonstrate that numerous CD11c + DCs in the AR mouse spleen underwent pyroptosis, while no obvious pyroptosis was observed in eosinophils or mast cells.

### Pyroptosis was significantly increased in CD11c + DCs in the spleens of AR mice, and NSA treatment attenuated CD11c + DC pyroptosis

**Fig. 2:**
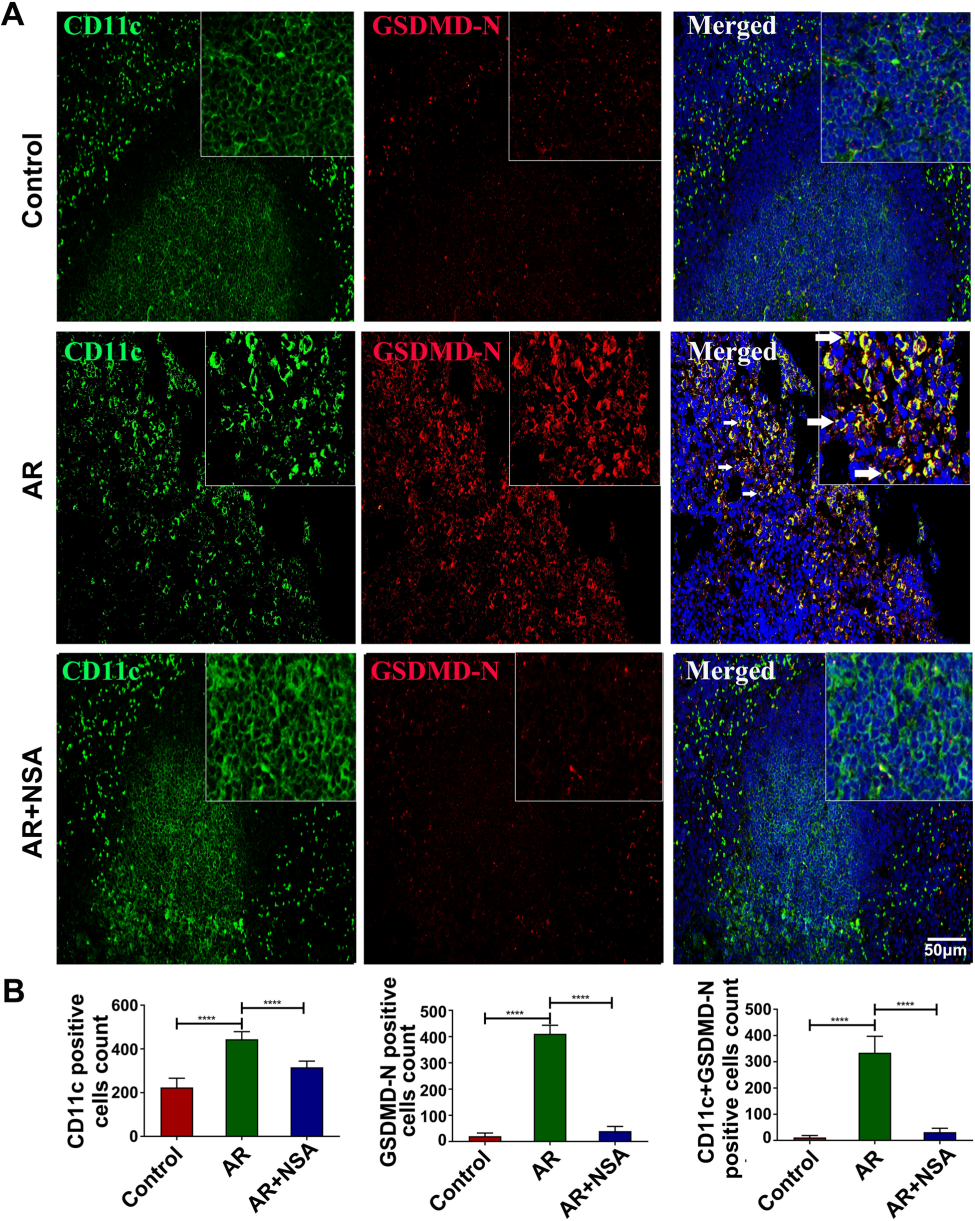
Pyroptosis was significantly increased in CD11c + DCs in the spleens of AR mice, and NSA treatment attenuated CD11c + DC pyroptosis. **A** ~ **B** Immunofluorescence analysis of CD11c-positive cells, GSDMD-N-positive cells and CD11c and GSDMD-N double-positive cells in the spleens of mice in each group. The arrows indicate CD11c and GSDMD-N double-positive cells. ****P < 0.0001

To study CD11c^+^ DC pyroptosis, GSDMD-N expression in CD11c^+^ DCs in mouse spleens was tested. Compared with those in the control group, the numbers of CD11c-positive cells, GSDMD-N-positive cells and CD11c and GSDMD-N double-positive cells in the spleens of AR mice were significantly increased (Fig. 2A ~ B; *P* < 0.05); however, these cells were decreased in the AR + NSA group compared to the AR group (Fig. 2A ~ B; *P* < 0.05). These results suggest that NSA inhibits the abnormal activation of CD11c + DC pyroptosis in the spleens of AR mice.

### Pyroptosis was significantly increased in CD11c ^+^ DCs in the nasal mucosa of AR mice, and NSA treatment attenuated CD11c^+^ DC pyroptosis

**Fig. 3:**
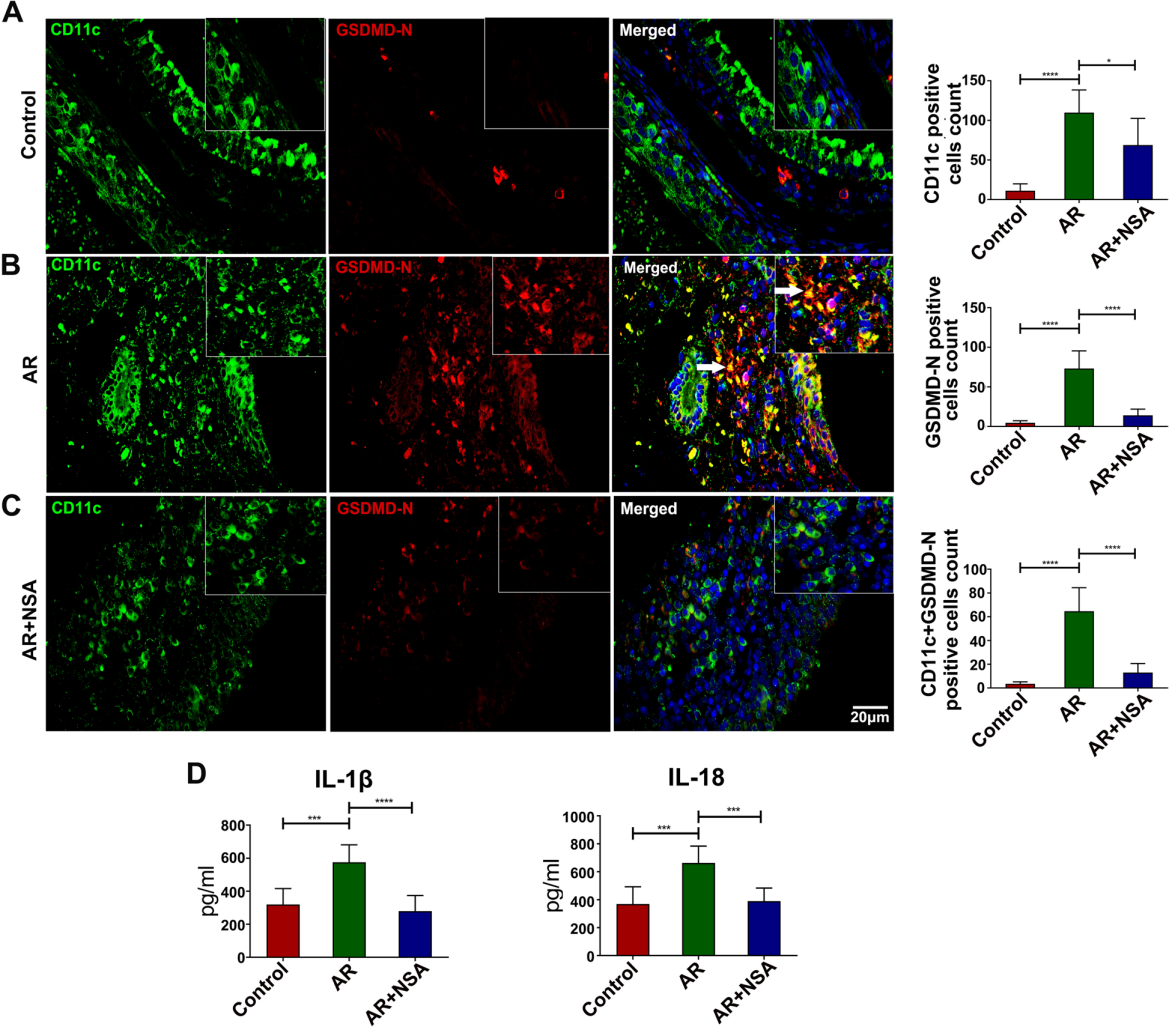
Pyroptosis was significantly increased in CD11c + DCs in the nasal mucosa of AR mice, and NSA treatment attenuated CD11c + DC pyroptosis. **A** Immunofluorescence analysis of CD11c-positive cells, GSDMD-N-positive cells and CD11c and GSDMD-N double-positive cells in the nasal mucosa of mice in the control group. **B** Immunofluorescence analysis of CD11c-positive cells, GSDMD-N-positive cells and CD11c and GSDMD-N double-positive cells in the nasal mucosa of mice in the AR group. **C** Immunofluorescence analysis of CD11c-positive cells, GSDMD-N-positive cells and CD11c and GSDMD-N double-positive cells in the nasal mucosa of mice in the AR + NSA group. The arrows indicate CD11c and GSDMD-N double-positive cells. **D** The serum expression levels of IL-1β and IL-18 in each group. *P < 0.05 ***P < 0.001 ****P < 0.0001

Because numerous DCs underwent pyroptosis in the spleens of AR mice, pyroptosis in the nasal mucosa was examined. Compared with those in the control group, the numbers of CD11c-positive cells, GSDMD-N-positive cells and CD11c and GSDMD-N double-positive cells in the nasal mucosa of AR mice were significantly increased (Fig. 3A ~ B; *P* < 0.05); however, all of these cells were decreased in the AR + NSA group compared to the AR group (Fig. 3B ~ C; *P* < 0.05).

In addition, compared to those in the control group, serum levels of IL-1β and IL-18 were increased in the AR group (Fig. 3D; *P* < 0.05). Compared with those in the AR group, mice treated with NSA exhibited markedly reduced levels of IL-1β and IL-18 (Fig. 3D; *P* < 0.05). These results suggest that NSA inhibits CD11c + DC pyroptosis in the nasal mucosa of AR mice and affects the subsequent inflammatory response.

### Pyroptosis was significantly increased in CD11c + DCs in the draining lymph nodes of the nose, and NSA treatment attenuated CD11c + DC pyroptosis

**Fig. 4:**
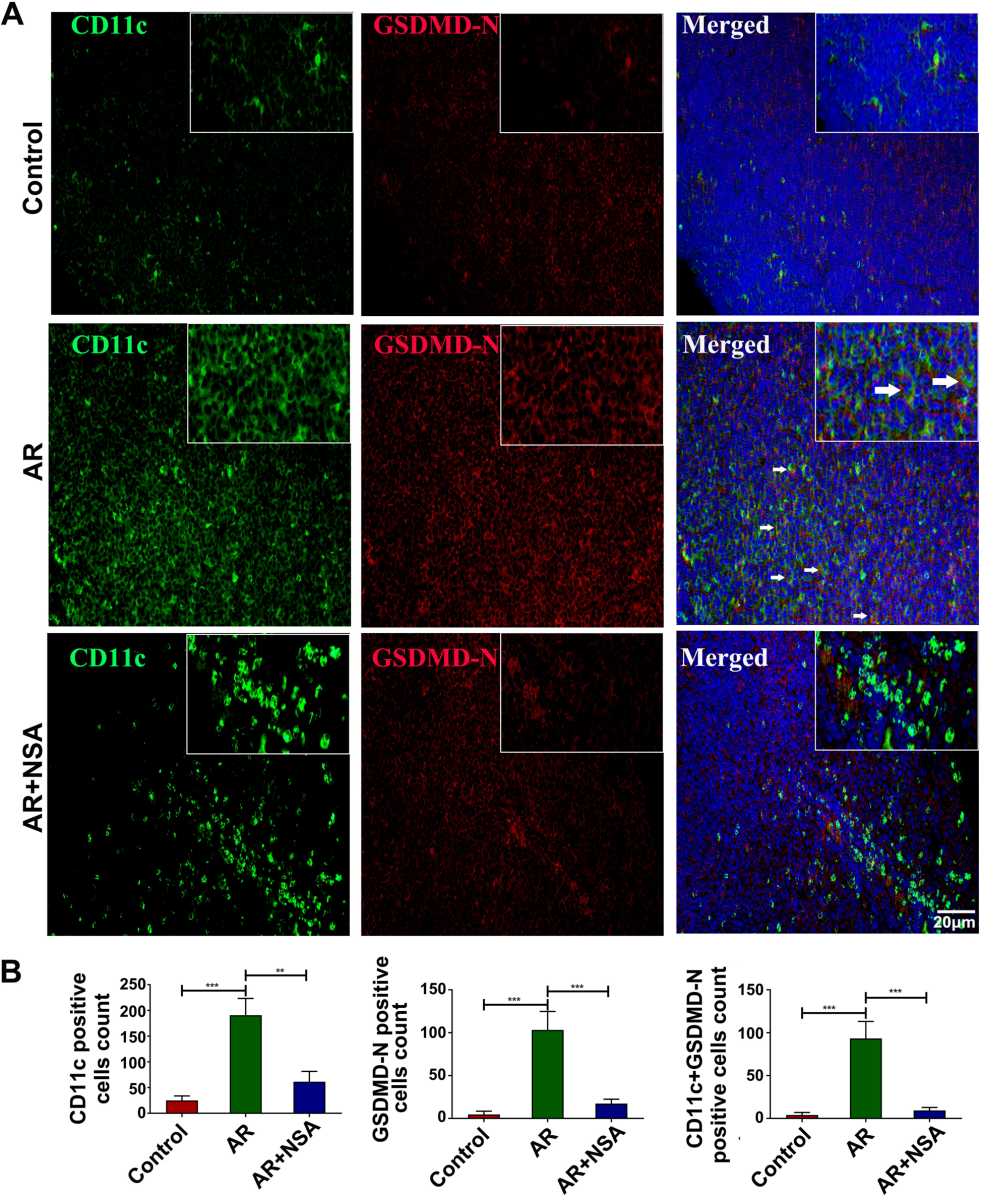
Pyroptosis was significantly increased in CD11c + DCs in the draining lymph nodes of the nose, and NSA treatment attenuated CD11c + DC pyroptosis. **A** ~ **B** Immunofluorescence analysis of CD11c-positive cells, GSDMD-N-positive cells and CD11c and GSDMD-N double-positive cells in the draining lymph nodes of the nose in each group. The arrows indicate CD11c and GSDMD-N double-positive cells. **P < 0.01, ***P < 0.001

To study CD11c^+^ DC pyroptosis, GSDMD-N expression in CD11c^+^ DCs in the draining lymph nodes of the nose was tested. Compared with those in the control group, the numbers of CD11c-positive cells, GSDMD-N-positive cells and CD11c and GSDMD-N double-positive cells in the draining lymph nodes of the nose in the AR group were significantly increased (Fig. 4A ~ B; *P* < 0.05); however, all of these cells were decreased in the AR + NSA group compared to the AR group (Fig. 4A ~ B; *P* < 0.05). These results suggest that NSA inhibits the abnormal activation of CD11c + DC pyroptosis in the draining lymph nodes of the nose.

### Inhibiting GSDMD-N-mediated pyroptosis alleviated the inflammatory responses in the AR model

**Fig. 5:**
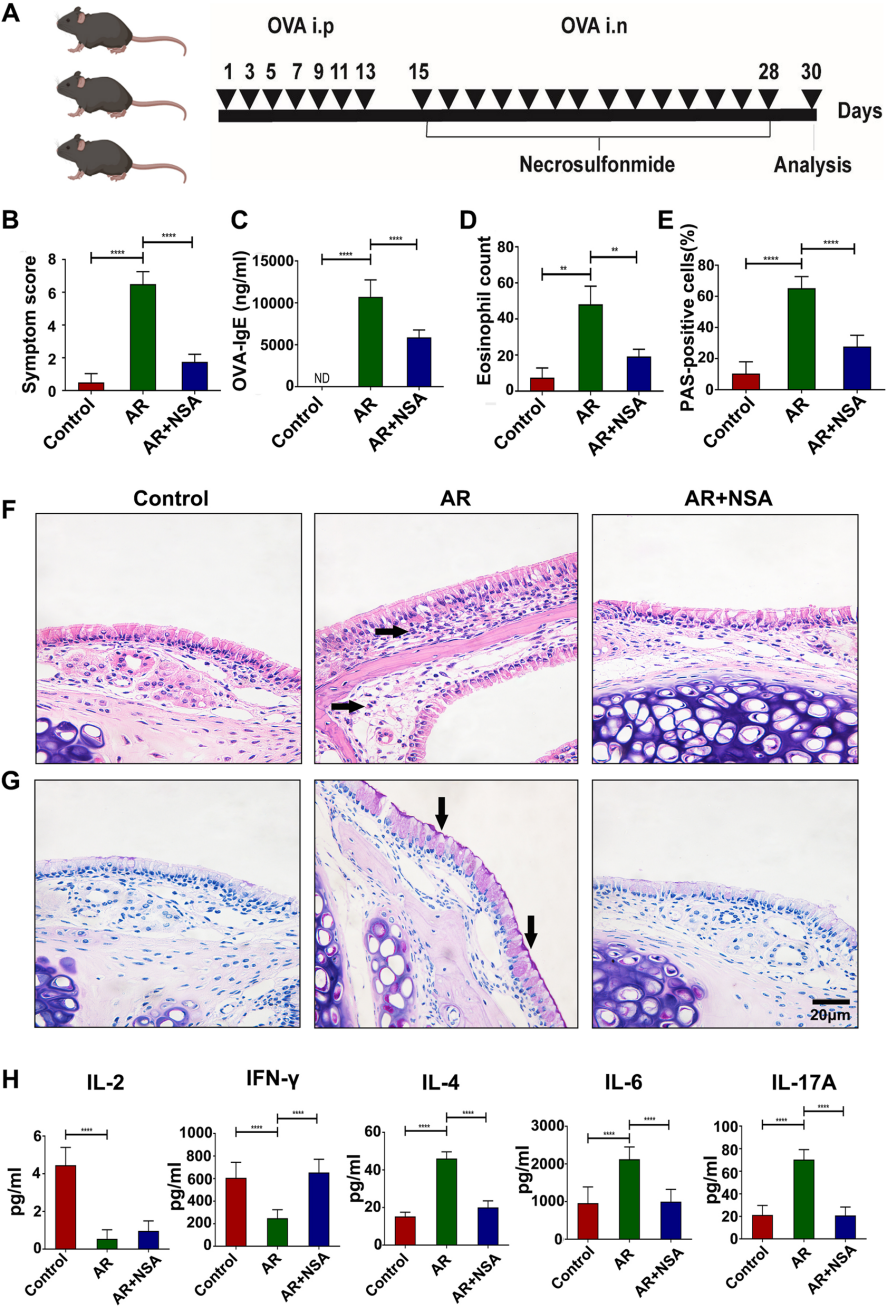
Inhibiting pyroptosis alleviated the inflammatory response in the AR model. **A** Experimental procedure. **B** Allergic symptom scores in each group. **C** OVA-specific serum IgE titres in each group. **D** Quantification of eosinophil infiltration. **E** Quantification of goblet cell hyperplasia. **F** Nasal mucosal sections were stained with haematoxylin and eosin (H&E), and the arrows indicate eosinophils (400X). **G** Nasal mucosal sections were stained with periodic acid–Schiff reagent (PAS), and the arrows indicate goblet cells (400X). **H** Statistical analysis of the concentrations of IL-2, IFN-γ, IL-4, IL-6 and IL-17 A in each group. The values represent the mean ± SD. *P < 0.05, **P < 0.01, ***P < 0.001, ****P < 0.0001. The data from three independent experiments are presented. AR, allergic rhinitis; NSA, necrosulfonamide

To investigate whether pyroptosis plays a role in the pathogenesis of AR, the mice were sensitized and challenged with ovalbumin (OVA) as previously described and then treated with regular injections of necrosulfonamide (NSA) (Fig. 5A). Compared with the control group, the AR group exhibited increased allergic symptom scores (Fig. 5B; *P* < 0.05), serum OVA-sIgE titres (Fig. 5C; *P* < 0.05), eosinophil infiltration, and goblet cell hyperplasia (Fig. 5D-G; *P* < 0.05), indicating the reliability of the AR model. Notably, compared with those in the AR group, mice in the AR + NSA group exhibited markedly reduced allergic symptom scores, serum OVA-sIgE titres, nasal eosinophil infiltration, and mucus hypersecretion (Fig. 5B-G; *P* < 0.05). These results strongly indicate that NSA can suppress OVA-induced allergic inflammation.

The effect of inhibiting GSDMD-N-mediated pyroptosis on Th1/Th2/Th17 cytokines in AR mice was investigated. The levels of proinflammatory cytokines, including Th2 (IL-4, IL-6) and Th17 (IL-17 A) cytokines (Fig. 5H, *P* < 0.05), were significantly increased by OVA stimulation, whereas Th1 (IL-2 and IFN-γ) cytokine levels were decreased (Fig. 5H, *P* < 0.05). Compared with the effects in the AR group, therapeutic administration of NSA markedly protected against a Th1/Th2/Th17 imbalance, as indicated by increased Th1 (IL-2 and IFN-γ) cytokine levels and decreased Th2 (IL-4, IL-6) and Th17 (IL-17 A) cytokine levels (Fig. 5H, *P* < 0.05). These data confirmed that abnormal activation of pyroptosis occurred in the AR model, and inhibiting GSDMD-N-mediated pyroptosis could significantly alleviate the inflammatory response.

### OVA/HDM stimulation increased pyroptotic morphological abnormalities and expression of pyroptosis-related proteins in BMDCs in a dose-dependent manner

**Fig. 6:**
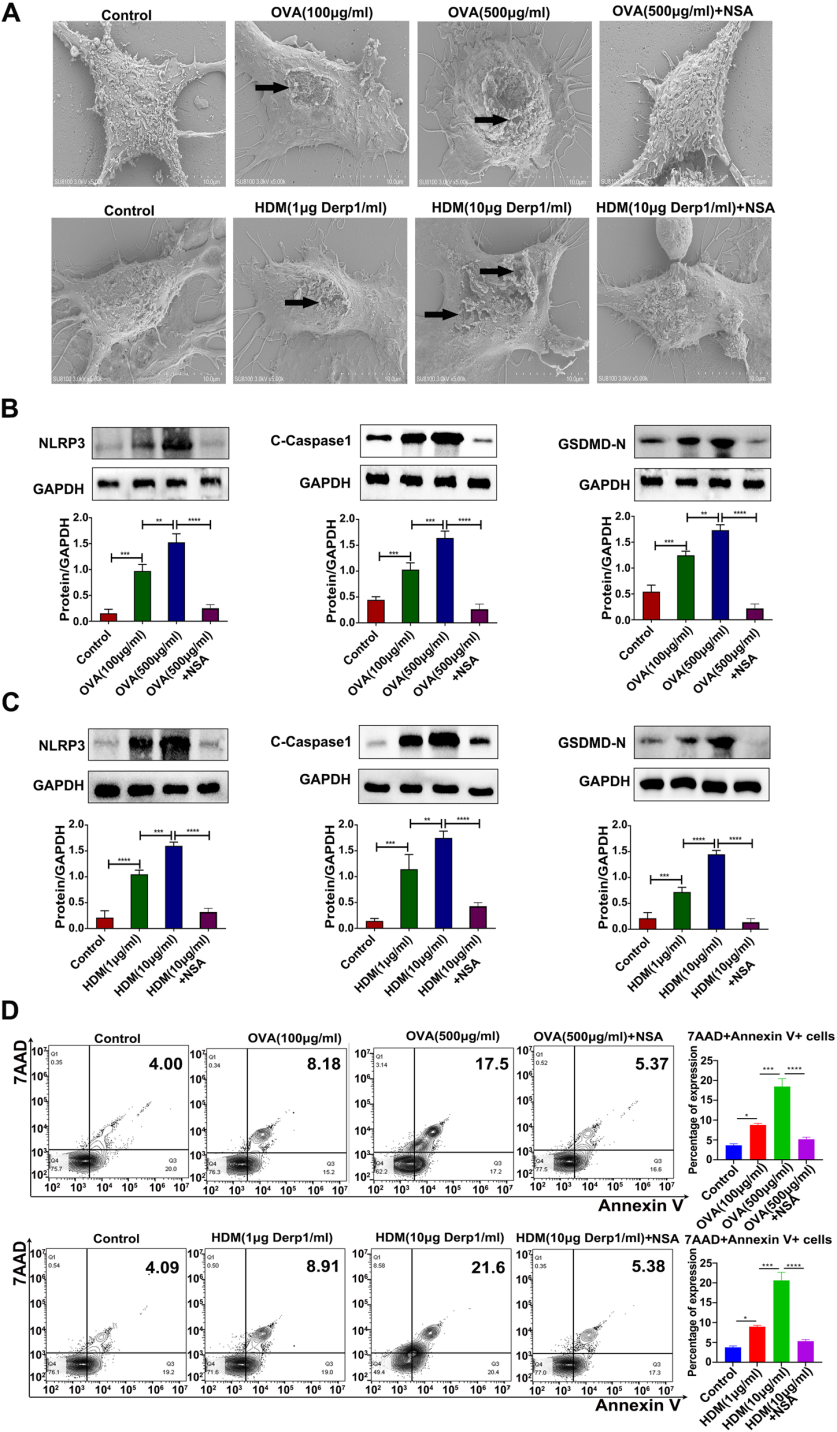
OVA/HDM stimulation increased pyroptotic morphological abnormalities and expression of pyroptosis-related proteins in BMDCs in a dose-dependent manner. **A** Representative scanning electron micrograph images of BMDCs. The arrow points to pyroptotic morphological abnormalities (8000 X) in BMDCs. **B** ~ **C** The expression of NLRP3, C-Caspase1 and GSDMD-N in each group. **D** Cell death was detected by flow cytometry. The data were collected from three independent experiments, with values representing the mean; *P < 0.05,**P < 0.01, ***P < 0.001, ****P < 0.0001

To investigate the direct effect of OVA/HDM on pyroptosis in BMDCs, BMDCs were induced in vitro. Compared with control treatment, OVA/HDM stimulation led to abnormal pyroptotic morphology in BMDCs, including numerous pores or pits of different sizes on the cell membrane, cell structural collapse, and cell membrane disruption, which increased with increasing OVA/HDM concentration (Fig. 6A). However, the abnormal pyroptotic morphology in BMDCs stimulated with 500 µg/mL OVA or 10 µg/ml HDM was significantly reduced by the addition of NSA (Fig. 6A).

The western blot results showed that compared with those in the control group, increasing OVA/HDM concentrations gradually increased the pyroptosis proteins NLRP3, C-Caspase1 and GSDMD-N. The highest expression was observed in the high-concentration group (Fig. 6B-C, *P* < 0.05). Moreover, after NSA-mediated inhibition of pyroptosis, the expression of NLRP3, C-Caspase1 and GSDMD-N decreased significantly compared with that in the OVA/HDM group (Fig. 6B-C, *P* < 0.05). These results confirmed that OVA/HDM induced pyroptosis in BMDCs in a dose-dependent manner and that NSA effectively blocked allergen-induced pyroptosis in BMDCs.

Compared with those in the control group, Annexin V + 7-AAD + double-positive cells gradually increased with increasing OVA/HDM concentrations (Fig. 6D, *P* < 0.05). Compared with those in the high-concentration OVA/HDM group, Annexin V + 7-AAD + double-positive cells were significantly reduced in the high-concentration OVA/HDM + NSA group (Fig. 6D, *P* < 0.05). Moreover, after NSA-mediated inhibition of pyroptosis, the number of Annexin V + 7-AAD + double-positive cells decreased significantly compared with that in the OVA/HDM group. These results confirmed that the number of dead DCs gradually increased with increasing OVA/HDM concentrations. The use of NSA can effectively block DC death.

### OVA induced BMDC pyroptosis by increasing the surface expression of MHC II molecules

**Fig. 7:**
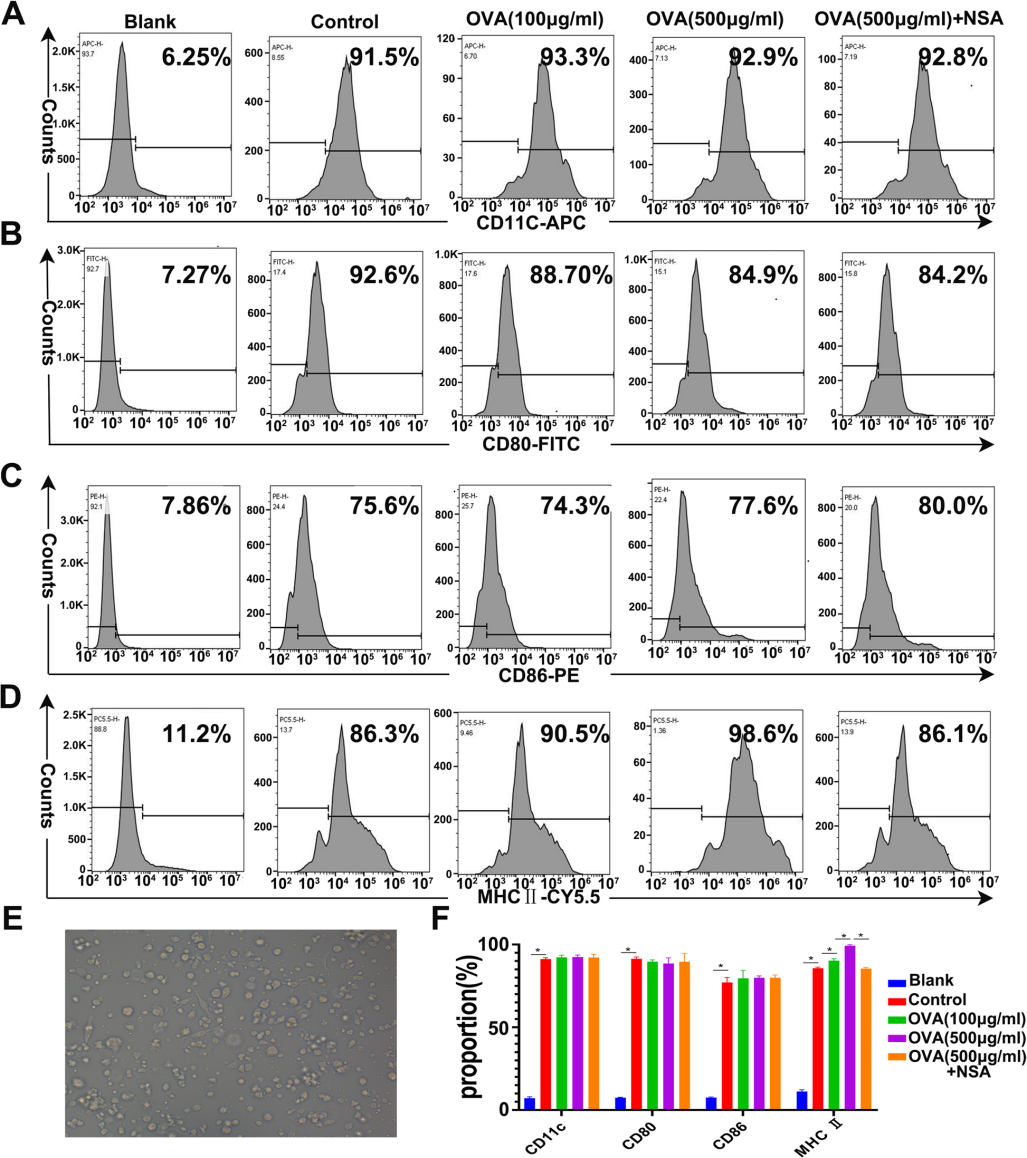
OVA induced BMDC pyroptosis by increasing the surface level of MHC II molecules. **A** Expression of CD11c on the cell surface in each group. **B** Expression of CD80 on the cell surface in each group. **C** Expression of CD86 on the cell surface in each group. **D** Expression of MHC II on the cell surface in each group. **E** Mature CD11c + BMDCs (200×) were observed under a microscope. **F** Statistical analysis of CD11c, CD80, CD86, and MHC II. The data were collected from three independent experiments, and the value represents the mean; independent t test, *P < 0.05

To study the effect of BMDC pyroptosis on the AR inflammatory response, we stimulated cultured CD11c + BMDCs with OVA and studied antigen presentation. In the blank group without the addition of the cytokines GM-CSF, IL-4, and TNF-α, the bone marrow cells almost failed to differentiate into CD11c + BMDCs (Fig. 7A). However, in the control group, which was treated with several cytokines, including GM-CSF, IL-4, and TNF-α, 90% of the bone marrow cells were converted into CD11c + BMDCs (Fig. 7A, *P* < 0.05). Similarly, 90% of the bone marrow cells in the low- and high-concentration groups and the OVA + NSA group were converted into CD11c + BMDCs (Fig. 7A, *P* < 0.05) with normal morphology (Fig. 7E). These results confirmed that we successfully induced CD11c + BMDCs in vitro.

To further verify the influence of OVA and NSA on the maturity and function of BMDCs, we examined BMDC surface expression of CD80, CD86, and several types of MHC II molecules. CD80 and CD86 reflect the maturity of DCs [24], and MHC II reflects the antigen-presenting function of DCs [25]. We found no significant difference in the expression of CD80 or CD86 on the CD11c + BMDC surface in the control group, low-concentration group, high-concentration OVA group, and OVA + NSA group (Fig. 7B, C, F, *P* < 0.05), suggesting that OVA-induced pyroptosis did not affect the maturation of CD11c + BMDCs. However, MHC II expression on the surface of CD11c + BMDCs was dose-dependently increased after the addition of OVA (Fig. 7D, F, *P* < 0.05). Compared with that in the high-concentration OVA group, MHC II expression was significantly decreased in the OVA + NSA group (Fig. 7D, F, *P* < 0.05). These results confirmed that a high concentration of OVA could induce pyroptosis in CD11c + BMDCs and promote BMDC antigen presentation, which could be blocked by pyroptosis inhibitors.

### Effects of BMDC pyroptosis on Th1/Th2/Th17 cell differentiation and the allergic inflammatory response

**Fig. 8:**
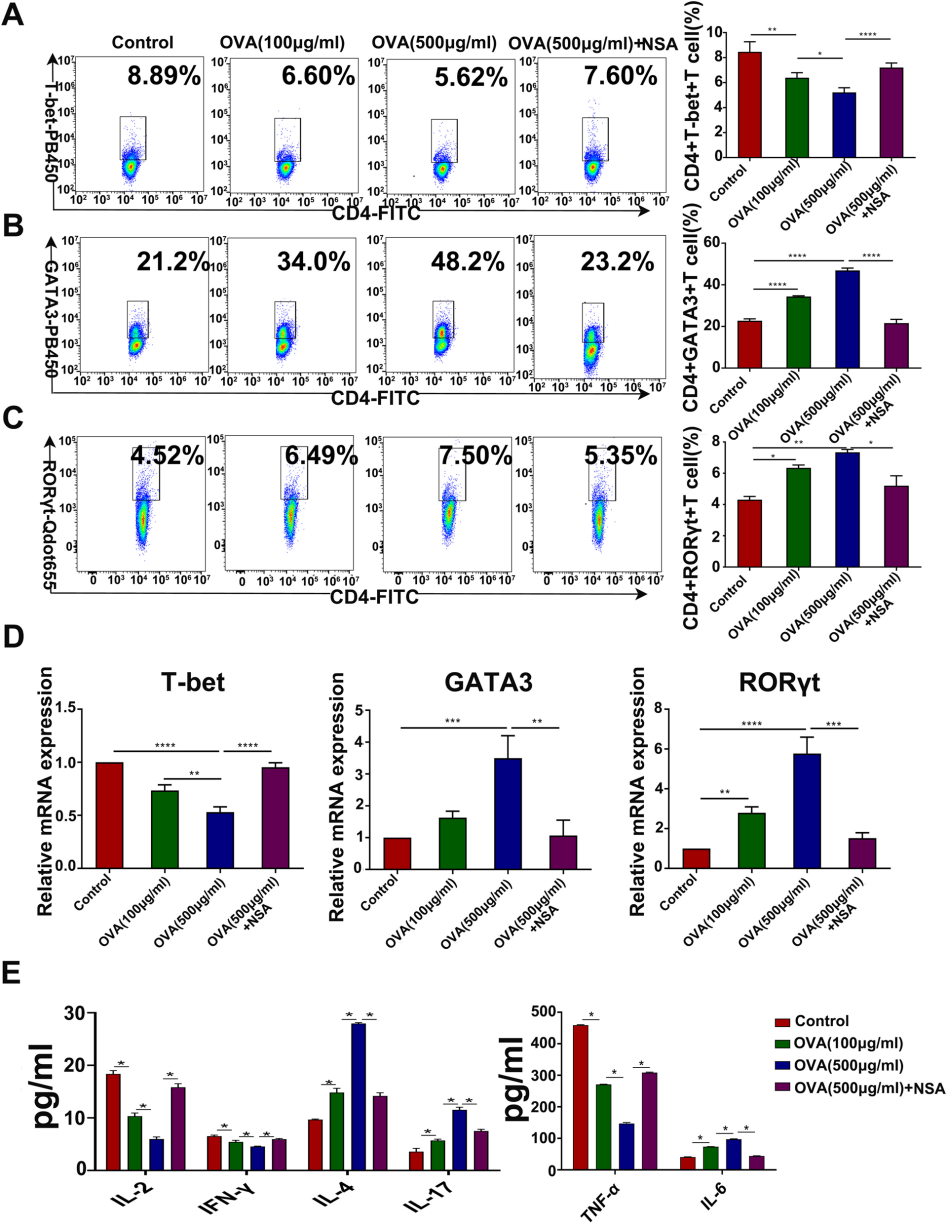
Effects of BMDC pyroptosis on Th1/Th2/Th17 cell differentiation during the allergic inflammatory response. **A**-**C** The numbers of Th1 cells (CD4 + T-bet+), Th2 cells (CD4 + GATA3+) and Th17 cells (CD4 + RORγt+) in the lymphocyte populations in each group. **D** ~ **F** Gene expression of T-bet, GATA3, and RORγt in the cocultures was determined by quantitative real-time PCR. **G** ~ **H** Levels of the cytokines IL-2, IFN-γ, IL-6 and IL-17 A in the supernatant of cells cocultured with lymphocytes in each group. *P < 0.05, **P < 0.01, ***P < 0.001, ****P < 0.0001

Next, the effects of OVA-induced BMDC pyroptosis on the differentiation of CD4 + T cells and the allergic inflammatory response were studied. The numbers of Th1 cells (CD4 + T-bet+) in the low- and high-concentration OVA groups were decreased compared with those in the control group (Fig. 8A, D, *P* < 0.05), while the numbers of Th2 cells (CD4 + GATA3+) and Th17 cells (CD4 + RORγt+) were increased (Fig. 8B-C, *P* < 0.05). Compared with those in the OVA group, the numbers of Th1 cells were increased and the numbers of Th2 and Th17 cells were decreased in the OVA + NSA group (Fig. 8A-C, *P* < 0.05).

The RT‒PCR results showed the same changes in key transcription factors of Th1/Th2/Th17 cells in the groups. Briefly, compared with that in the control group, the expression of T-bet was decreased, and the expression of GATA3 and RORγt was increased in the low- and high-concentration OVA groups. Moreover, markedly increased T-bet levels, as well as significantly decreased GATA3 and RORγt expression, were observed in the OVA + NSA group (Fig. 8D ~ F, *P* < 0.05). These results suggest that pyroptosis inhibitors can effectively block pyroptosis in BMDCs and the induction of allergic inflammation by reversing the immune imbalance of CD4 + T-cell subtypes.

Concomitantly, the levels of Th1 (IL-2, TNF-α, and IFN-γ) cytokines in lymphocyte coculture supernatants were dose-dependently decreased in the OVA group, while the levels of Th2 (IL-4, IL-6) and Th17 (IL-17 A) cytokines were significantly increased in a dose-dependent manner (Fig. 8G ~ H, *P* < 0.05). Compared with those in the OVA group, the levels of Th1 (IL-2, TNF-α, and IFN-γ) cytokines in the coculture supernatant were increased, and Th2 (IL-4, IL-6) and Th17 (IL-17 A) cytokines were decreased in the OVA + NSA group (Fig. 8G ~ H, *P* < 0.05). These results suggest that BMDC pyroptosis can directly induce an allergic inflammatory response by promoting Th1/Th2/Th17 cytokine imbalance.

### Adoptive transfer of OVA-loaded BMDCs could significantly promote allergic inflammation, while the administration of NSA to OVA-loaded BMDCs could significantly reduce AR inflammation

**Fig. 9:**
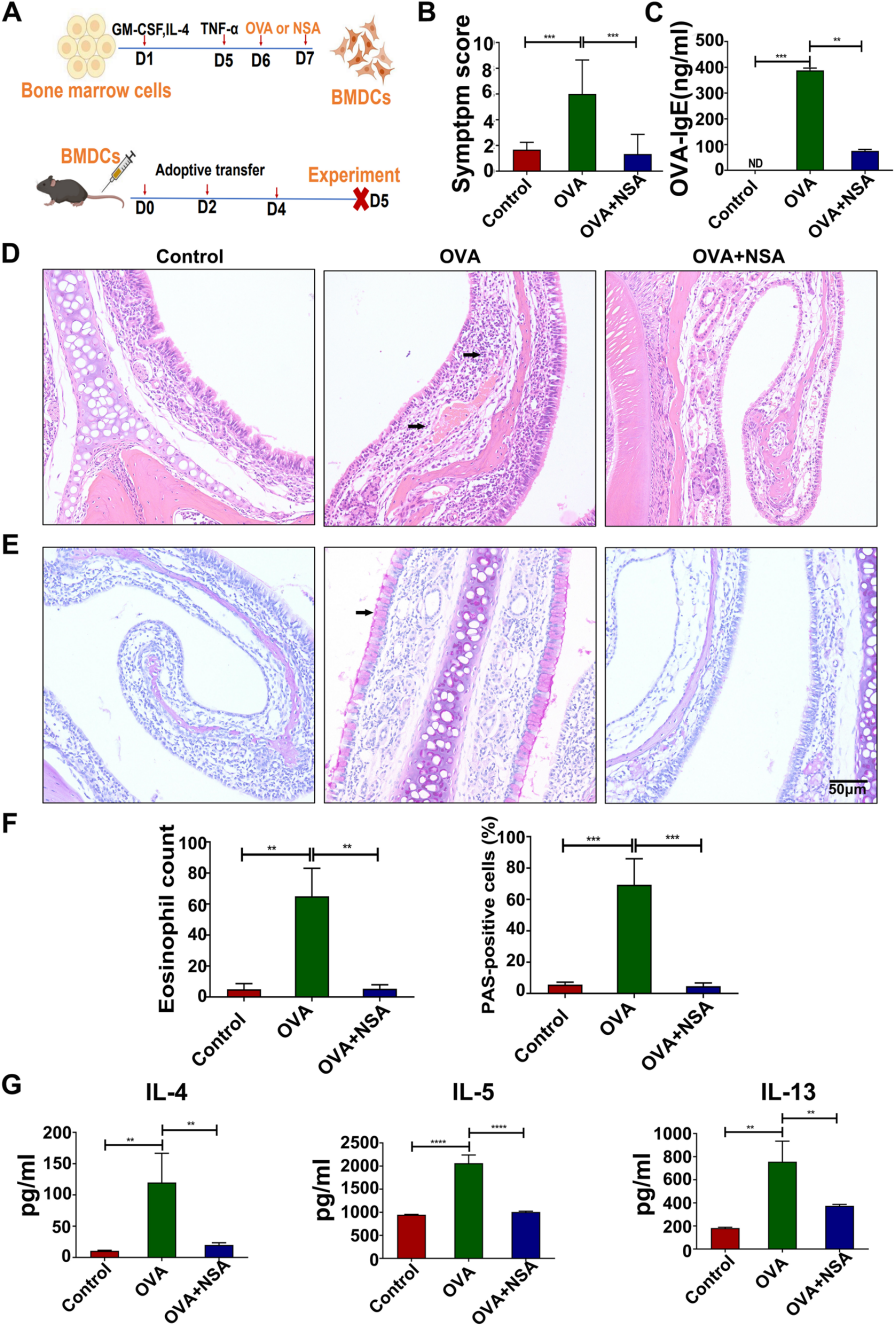
Adoptive transfer of OVA-loaded BMDCs significantly promoted allergic inflammation, while the administration of NSA to OVA-loaded BMDCs significantly reduced AR inflammation. **A** Experimental protocol for the construction of an adoptive transfer animal model. **B** Symptomatic scores in each group. **C** Serum levels of OVA-sIgE in each group. **D** Histopathological changes in the nasal mucosa; the arrow indicates eosinophils (400X). **E** Proliferation of goblet cells in the nasal mucosa; the arrow indicates proliferating goblet cells (200X). **F** Eosinophils or proliferating goblet cells were counted at a high magnification. **G** Levels of the cytokines IL-4, IL-5, and IL-13 in each group. *P < 0.05 and **P < 0.01 ***P < 0.001 ****P < 0.0001

Since OVA induced BMDC pyroptosis in a dose-dependent manner, an adoptive transfer murine model was further used to study the role of BMDC pyroptosis in allergic inflammation (Fig. 9A). Compared with those in the control group, mice in the OVA group exhibited increased allergic symptom scores (Fig. 9B; *P* < 0.05), OVA-specific serum IgE titres (Fig. 9C; *P* < 0.05), eosinophil infiltration, and goblet cell hyperplasia (Fig. 9D-F; *P* < 0.05). However, compared with those in the OVA group, mice in the OVA + NSA group exhibited markedly reduced allergic symptom scores, serum OVA-specific IgE titres, nasal eosinophil infiltration, and mucus hypersecretion (Fig. 9B-F; *P* < 0.05). Furthermore, compared with those in the control group, the levels of the cytokines IL-4, IL-5 and IL-13 were significantly increased in the OVA group. The administration of NSA to OVA-loaded BMDCs significantly reversed the cytokine imbalance (Fig. 9G; *P* < 0.05). These results further indicated the important role of DC pyroptosis in the development of AR.

## Discussion

AR results from a failure to develop tolerance towards a specific allergen, resulting in the emergence of an allergen-specific Th2 response [22, 26]. Pyroptosis is also involved in the development and progression of allergic diseases. In addition, an OVA-induced AR model was established. Notably, the expression levels of IL-1β and IL-18 were upregulated in AR mice. Further analysis showed that the abundance of pyroptotic cells was increased in the AR group and that pyroptosis mainly occurred in CD11c + DCs rather than basophils or mast cells. Additionally, CD11c + BMDCs were cultured in vitro, and the expression levels of pyroptosis-related proteins were significantly elevated in BMDCs stimulated with OVA, suggesting that BMDC pyroptosis was induced by OVA stimulation in vitro. Therefore, we demonstrated that GSDMD-N-mediated CD11c + DC pyroptosis was abnormally activated in AR.

NSA, a GSDMD inhibitor [14, 15], was used to monitor pyroptosis-related protein levels and inflammatory changes in AR mice. The expression levels of GSDMD-N, IL-1β, and IL-18 were significantly decreased after NSA administration, followed by decreased pyroptosis in CD11c + DCs in the nasal mucosa and spleens of mice. Additionally, mice treated with NSA exhibited markedly reduced allergic symptom scores, serum OVA-specific IgE titres, nasal eosinophil infiltration, and mucus hypersecretion. We confirmed that allergen-induced CD11c + DC pyroptosis plays an important role in the pathogenesis of AR.

Th1-type cytokines (IFN-γ, IL-2), Th2-type cytokines (IL-4, IL-6), and Th17-type cytokines (IL-17 A) have been previously verified [27, 28], and we found that NSA ameliorated the Th1/Th2/Th17 imbalance, which was typically altered in subjects with allergen-induced inflammation. Briefly, the levels of the proinflammatory cytokines IL-4, IL-6 and IL-17 A were significantly decreased in response to NSA administration, whereas the levels of the Th1 cytokines IFN-γ and IL-2 were increased. These results provided definitive evidence that inhibiting GSDMD-N-mediated pyroptosis markedly protected against a Th1/Th2/Th17 imbalance and alleviated allergic inflammation in the AR model.

IL-1β and IL-18 can stimulate the differentiation and proliferation of Th2 cells and exacerbate related allergic reactions [29, 30]. IL-18 synergizes with IL-23 to induce IL-17 secretion by Th17 cells [31]. Additionally, in combination with IL-6 and IL-23, IL-1β plays a role in the development of Th17 cell responses. In this study, the pyroptosis inhibitor NSA blocked GSDMD-N-mediated pyroptosis, and we speculated that NSA inhibited the release of IL-1β and IL-18 and reversed the Th1/Th2/Th17 immune imbalance.

Moreover, the effects of allergen-induced BMDC pyroptosis on downstream effector T cells were examined. After OVA-stimulated BMDCs were cocultured with lymphocytes [32], the numbers of Th1 cells were decreased, and the numbers of Th2 and Th17 cells were increased, followed by the downregulation of IL-2 levels and upregulation of the proinflammatory cytokines IL-4 and IL-17 A. The Th1/Th2/Th17 cell imbalances were significantly reversed by the addition of NSA. This is similar to the result reported by Yoshimoto et al. [33, 34] that IL-1β and IL-18 could promote the differentiation of proinflammatory CD4 + T cells, such as Th2/Th17 cells [35]. On the other hand, we speculated that BMDC pyroptosis could enhance antigen presentation capacity [36]. OVA induced BMDC pyroptosis in a dose-dependent manner, and the expression of MHC II molecules on the surface of BMDCs increased with increasing OVA concentrations, suggesting that BMDC pyroptosis could enhance antigen presentation, resulting in an imbalance in Th1/Th2/Th17 cells. These results confirmed that OVA-induced BMDC pyroptosis resulted in the abnormal differentiation of downstream effector T cells, thereby inducing a subsequent allergic inflammatory response. The mechanism is related to the release of IL-1β and IL-18 or the enhancement of antigen presentation during pyroptosis.

In addition, an adoptive transfer murine model was further used to study the role of BMDC pyroptosis in allergic inflammation [37, 38]. Interestingly, i.v. injection of OVA-loaded BMDCs alone 3 times in the absence of i.p. OVA sensitization elicited an allergic phenotype after 5 days. The administration of NSA to OVA-loaded BMDCs could significantly reduce allergic symptom scores, serum OVA-specific IgE titres, nasal eosinophil infiltration, and mucus hypersecretion. These results further indicated the important role of DC pyroptosis in AR inflammation.

In conclusion, the number of DCs increased significantly after OVA stimulation. Pyroptosis occurred in some DCs and induced subsequent allergic inflammation. This part of the inflammatory response was independent of DC antigen presentation and the induction of the allergic inflammatory response. This result provides potential clues for allergic disease interventions.

## Data Availability

The data that support the fndings of this study are available from the corresponding author upon reasonable request.
